# Exploration of yeast biodiversity from Thai flowers and optimization of carotenoid production by a promising isolate

**DOI:** 10.1093/jimb/kuag003

**Published:** 2026-01-08

**Authors:** Pirapan Polburee, Thippawan Kodpan, Krittawan Tondee, Nontakorn Wimoolchat

**Affiliations:** Department of Microbiology, Faculty of Science, Srinakharinwirot University, 114 Sukhumvit 23, Bangkok 10110, Thailand; Department of Microbiology, Faculty of Science, Srinakharinwirot University, 114 Sukhumvit 23, Bangkok 10110, Thailand; Department of Microbiology, Faculty of Science, Srinakharinwirot University, 114 Sukhumvit 23, Bangkok 10110, Thailand; Department of Microbiology, Faculty of Science, Srinakharinwirot University, 114 Sukhumvit 23, Bangkok 10110, Thailand

**Keywords:** carotenoids, red yeast, *Rhodotorula*, fermentation optimization, yeast biodiversity

## Abstract

Microbial synthesis of carotenoids has garnered significant attention as an eco-friendly alternative to conventional synthetic methods and its facile extraction for impressive yield. This study delves into the efficacy of carotenoid production from a red yeast strain, as well as the biodiversity of yeast species from Thai flowers. The research involved the collection of flower samples within Thailand, along with 12 yeast species from 10 genera of Ascomycetes and 4 genera of Basidiomycetes which were isolated by identifying the D1/D2 domain of the large subunit rRNA gene. Unexpectedly, *Rhodotorula paludigena* SWU-FKT03 emerged as the top performing yeast strain, boasting an impressive carotenoid production rate of 183.30 ± 5.00 mg/L among the 36 red yeast strains isolated. Subsequently, a further investigation was performed, focusing on optimized culture conditions for carotenoid production from this yeast strain. The results were promising, as carotenoid production surged to 288.27 mg/L when 20 g/L of glucose and 10 g/L of monosodium glutamate served as the carbon and nitrogen sources, respectively. These findings underscore the potential of the *R. paludigena* SWU-FKT03 as a high-yield carotenoid producer when cultivated in shaking flasks, exhibiting a three-fold increase in carotenoid content when under optimized conditions. These results hint at the potential of this approach for future large-scale carotenoid production.

**One-sentence summary** A novel red yeast, *Rhodotorula paludigena* SWU-FKT03, isolated from Thai floral ecosystems, demonstrated high-yield carotenoid production of 288.27 mg/L after fermentation optimization, establishing a significant potential for industrial applications.

## Graphical Abstract

**Graphical Abstract:**
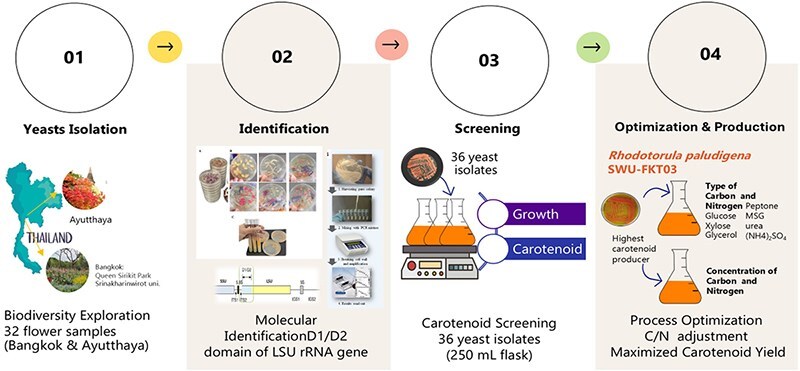

## Introduction

Carotenoids are a class of high-value natural pigments widely utilized in the food, feed, pharmaceutical, and cosmetic industries. Their commercial importance is attributed not only to their vibrant colors but also to their significant bioactive properties, including potent antioxidant activity, pro-vitamin A function, and potential role in reducing the risk of certain chronic diseases (Mata-Gómez et al., 2014). Driven by a strong consumer preference for natural over synthetic additives, the global market for carotenoids is expanding rapidly, creating a demand for sustainable and economically viable production methods (Novoveská et al., 2019). While chemical synthesis routes are established, they often involve high costs and do not meet the ‘‘natural label’’ threshold. Conversely, extraction from agricultural sources is constrained by geographical and seasonal variability, land-use requirements, and often results in insufficient yields for industrial needs (Fraser & Bramley, 2004).

Microbial fermentation provides a controllable, scalable, and sustainable platform for carotenoid production. Among microbial candidates, pigmented red yeasts (Basidiomycetous yeasts), particularly from the genera *Rhodotorula, Rhodosporidium*, and *Sporobolomyces*, are considered promising ‘‘cell factories’’ (Li et al., 2017). Their ability to co-produce high-value metabolites like lipids (single-cell oils) and enzymes, and the advancements in genetic engineering tools such as CRISPR/Cas9 systems developed for *Rhodotorula* species. (Guerfali, 2025; Mussagy et al., 2023; Tong et al., 2025). These yeast strains are known to naturally accumulate significant intracellular quantities of commercially relevant carotenoids, such as β-carotene, torulene, and torularhodin. Furthermore, they can often metabolize a wide range of low-cost carbon substrates, are robust to fermentation conditions, and certain species are generally recognized as safe, facilitating their application in food products (Zhang et al., 2024). However, to achieve commercial competitiveness, the yield and productivity of wild-type strains must be enhanced. This necessitates a continued bioprospecting effort to discover novel isolates with inherently superior production capabilities from unique ecological niches.

The floral ecosystem represents a compelling and under-explored environment for isolating novel yeast strains. The high-sugar content of nectar provides a rich carbon source, while constant exposure to solar UV radiation may act as a strong selective pressure for microorganisms that produce photoprotective pigments like carotenoids (Sofrona et al., 2020). Thailand, located in a tropical biodiversity hotspot, possesses a vast and largely uncharacterized diversity of flowering plants, presenting a unique opportunity for the bioprospecting of robust, pigment-producing yeasts (Kanpiengjai et al., 2023; Rosa et al., 2023). Therefore, yeasts isolated from flowers of this region are not merely novel; they are effectively prescreened by nature to be robust and potent producers of bioactive compounds, making them prime candidates for bioprocess development (Brysch-Herzberg, 2004; Ghaderiardakani et al., 2017).

Discovering a potent strain is merely the first step toward industrial application. To unlock the full metabolic potential of a promising isolate, a systematic optimization of its cultivation conditions is essential. Key nutritional and physical parameters including the type and concentration of carbon and nitrogen sources, the C/N ratio, initial pH, and temperature are known to profoundly influence both biomass accumulation and carotenoid biosynthesis (Beltrán & Wurtzel, 2025; Jo et al., 2025; Li et al., 2026; Xie et al., 2025; Zhao et al., 2025). Therefore, systematic process optimization is required to maximize final product titers and volumetric productivity, thereby bridging the gap between laboratory discovery and industrial feasibility.

The present study was thus designed with three primary objectives: (1) to explore the biodiversity of yeast by isolating strains from a wide variety of flowers in Thailand; (2) to select the most potent carotenoid-producing isolate; and (3) to systematically optimize the nutritional fermentation parameters to maximize carotenoid yield from the selected isolate. This work aims to contribute a novel, high-performance yeast strain for the biotechnology industry and establish an efficient bioprocess for the sustainable production of natural carotenoids.

## Materials and methods

### Yeast isolation

Flower samples were collected from the public park of Ayutthaya province and Queen Sirikit Park of Bangkok, Thailand. Yeast strains were isolated by placing the flower samples onto Yeast Extract Malt Extract (YM) agar medium (consisting of 3 g/L yeast extract, 3 g/L malt extract, 5 g/L peptone, 10 g/L glucose, and 20 g/L agar). To inhibit the growth of fungi and bacteria, the medium was supplemented with 250 mg/L sodium propionate and 200 mg/L chloramphenicol, incubated at 30°C until yeast colonies appeared. Yeast colonies of different morphologies were picked and purified by cross streaking on YM agar. Morphological characteristics of the isolated yeasts were examined under a light microscope at 400x magnification. Purified yeast cultures were maintained on YM agar at 4°C for short-term storage. For long-term preservation, the isolates were stored in YM broth containing 20% (v/v) glycerol at −80°C.

### Identification of the carotenoid yeast

The isolated yeast strains were categorized using molecular taxonomy in order to identify the carotenoid yeast by comparing the D1/D2 region of the large subunit (LSU) rRNA gene sequence. The PCR products were obtained from the strains’ genomic DNA using primers NL1 (5′-GCATATCAATAAGCGGAGGAAAAG-3′) and NL4 (5′-GGTCCGTGTTTCAAGACGG-3′) to determine the sequences of the D1/D2 region of the LSU rRNA gene (Kurtzman & Robnett, 1998). The LSU rRNA gene of the D1/D2 region of yeast was extracted and amplified using the procedure previously outlined in Limtong et al. (2007). The PCR product was purified using a Gel/PCR DNA Fragments Extraction Kit (Geneaid Biotech, Taiwan) and examined using agarose gel electrophoresis. Macrogen Inc. (Korea) received the purified product and used primers NL1 and NL4 to sequence the D1/D2 region of the LSU rRNA gene.

Identification of possibly distinct ascomycetous yeast species was then carried out using the widely recognized rule found in Kurtzman and Robnett (1998) study whereby strains exhibiting >1% nucleotide substitutions in the 600–500 nucleotides of the D1/D2 region are probably distinct species, while strains with 0–3 nucleotide differences are either conspecific or sister species. Likewise, Fell et al. (2000) recommendation, in which strains with two or more nucleotide divergences in the D1/D2 area indicated distinct species, and that strains within a species had similar sequences in this region, was used to identify possibly distinct strains of basidiomycetous yeast.

### Selection of high carotenoid production potential in red yeast

The yeast isolated represent the red colorogenic colonies and were selected for screening of carotenoid production, as these strains are characteristic of carotenoid-synthesizing yeasts. To prepare the inoculum, active yeasts were grown in a 250 mL Erlenmeyer flask with 30 mL of YPD medium (10 g/L yeast extract, 20 g/L peptone, and 20 g/L glucose) and incubated on a rotary shaker (Biobase BJPX-1102C, China) at 200 rpm and 30°C for 24 h. The inoculum was transferred to 50 ml of YPD medium in a 250 ml Erlenmeyer flask and incubated on a rotary shaker at 200 rpm and 30°C for 72 h. The initial OD at 600 nm was set to 1 in all the experiments undertaken, with triplicates of each selected isolate performed. Cells were harvested for biomass analysis and carotenoid concentration.

Yeast strains exhibiting the highest carotenoid-production were selected for further carotenoid cultivation in a 500 mL Erlenmeyer flask with 100 mL of YPD medium for 120 h under the same culture conditions. The sample was harvested every 24 h to determine growth and carotenoid production patterns at each stage of time.

### Optimization of high carotenoid production potential in red yeast

To enhance carotenoid production, the following optimization strategy was employed in a shake-flask cultivation: all experiments were conducted in 500 mL Erlenmeyer flasks containing 100 mL of YPD medium, incubated at 30°C with shaking at 200 rpm for 120 h. Samples were collected every 24 h to determine cell growth and carotenoid concentration. The optimization process of carbon, nitrogen and their optimum concentration were performed sequentially.

In order to determine the optimal source of carbon for carotenoid production, the standard carbon source (20 g/L glucose) in YPD medium (10 g/L yeast extract, 20 g/L peptone) was then replaced with either xylose or glycerol at the same concentration (20 g/L). The carbon source that yielded the highest carotenoid concentration was selected for subsequent experiments. Then the standard nitrogen source (20 g/L peptone) was replaced with various nitrogen compounds, including monosodium glutamate (MSG-10 g/L), urea (5.8 g/L), and ammonium sulfate (13 g/L) to likewise determine a possibly optimal source. Each new nitrogen source was added to provide a nitrogen concentration equivalent to that of 20 g/L peptone. The nitrogen source that yielded the highest carotenoid concentration was then selected for further optimization.

To optimize carbon source concentration, the best nitrogen source from previous experiments was used. The concentration of the optimal carbon source (glucose) was varied across a range of 10, 20, 30, and 40 g/L to determine the most effective concentration. Finally, the nitrogen source concentration was optimized using the optimal carbon concentration, the concentration of the best nitrogen source (MSG) was varied across a range of 5, 10, and 15 g/L.

Finally, the combination of the determined optimal carbon and nitrogen source concentrations were determined to be the optimal medium for yielding the highest carotenoid production of the selected yeast strain. Key kinetic parameters, including final titer, specific content, yield, and productivity, were then calculated from this final optimized condition.

### Analytical methods

The carotenoid was extracted from the 5 mL of culture, after the centrifuge, discarding the supernatant, then adding 2 mL of DMSO, 3 mL of acetone, and 20 g of glass beads The solution was then mixed to break up the cells for 2 min before undergoing another session in the centrifuge. Finally, the supernatant was measured at OD450nm by spectrophotometer (Shimadzu UV-1900i VIS, Japan) (Michelon et al., 2012). The carotenoid content was qualified by comparison of the β-carotene as the standard. The carotenoid composition was analyzed by Thin-layer chromatography using petroleum ether:acetone (4:1) as the mobile phase and silica gel as the stationary phase. The result was compared to the bata-carotene standard by analyzing the retention factor (*Rf*) values, which were calculated as the distance traveled by the substance divided by the distance traveled by the solvent.

Cell growth was monitored by measuring the optical density at 600 nm (OD_600_₀₀) using a spectrophotometer (Shimadzu UV-1900i VIS, Japan). To analyze the biomass, cells were harvested by the separation of cells using a centrifuge (Tomy MX 301, Japan) at 5,000 rpm for 5 min, washed with distilled water, and then dried at 100°C to a constant weight. The biomass was determined gravimetrically.

The experiment was carried out in triplicate with the same initial cell concentration at 1 of OD_600nm_. Results are expressed as mean ± standard deviation (SD). The effects of the condition on the carotenoid content were evaluated using a one-way ANOVA, and pairwise comparisons were then conducted using the Tukey post-hoc test (statistical significance was established at *p* < .05).

## Results and discussion

### The diversity of yeast from flowers

**Figure 1:**
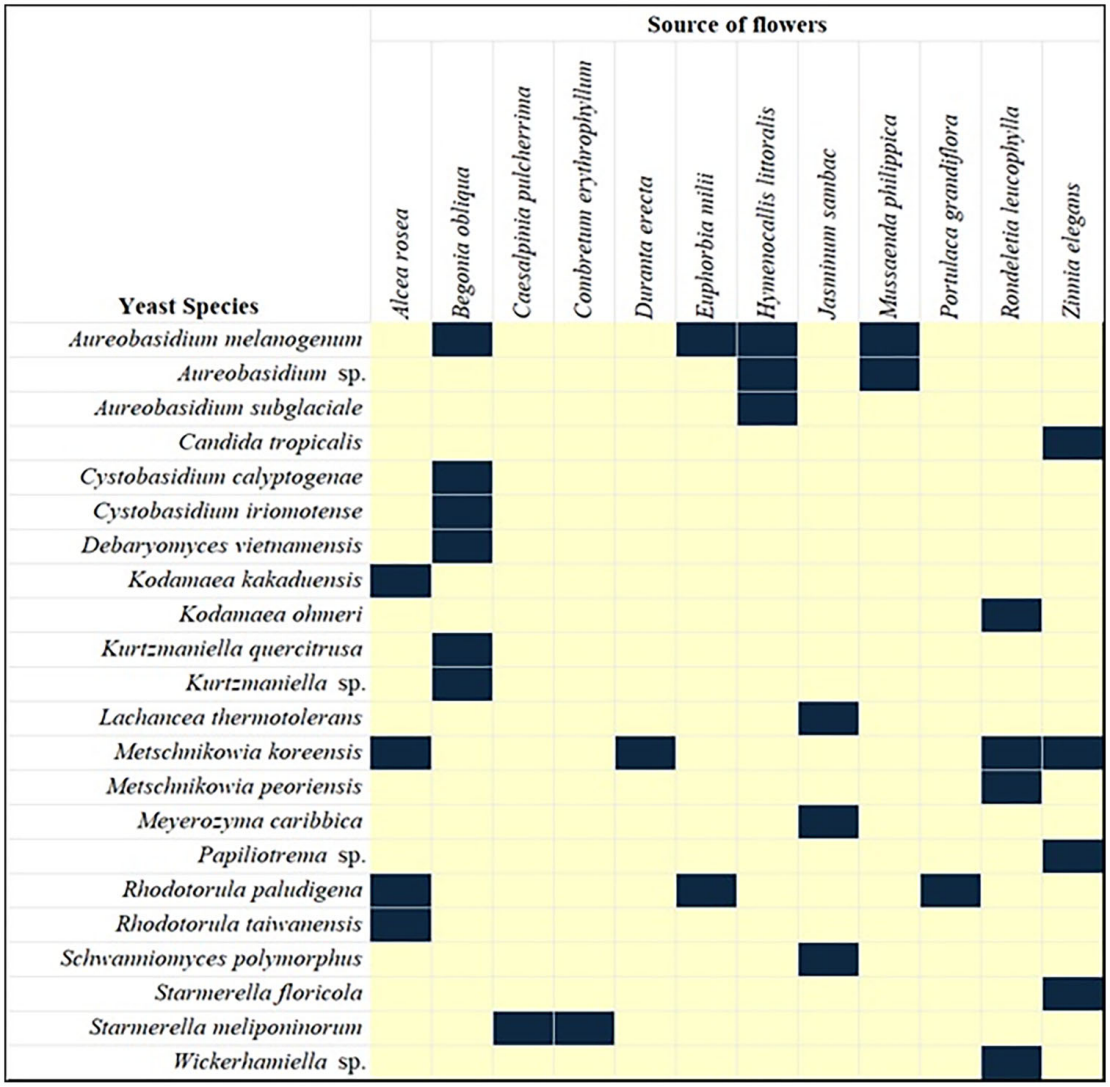
The association between yeast species isolated and their corresponding floral sources from various plant species in Thailand. The presence of each yeast species on a specific flower is indicated by a dark-shaded cells (■), while unshaded cells (□) denote absence.

The 12 species of flowers were collected from Ayutthaya province and Bangkok, Thailand. The yeast strains were isolated from flower samples by the direct plating method on YM medium (Supplementary Fig. S1). A total of 93 yeast isolates were obtained and identified by comparing the D1/D2 region of the LSU rRNA gene sequence. The results represent 10 genera of Ascomycetes with 16 species including *Starmerella floricola, Candida tropicalis, Metschnikowia koreensis, Meyerozyma caribbica*, and *Schwanniomyces polymorphus*; and Basidiomycetes with 4 genera and 6 species including *Papiliotrema* sp., *Rhodotorula paludigena, Rhodotorula taiwanensis, Wickerhamiella* sp., and *Cystobasidium calyptogenae*. Based on these results, two strains were identified as *Aureobasidium* sp. and *Papiliotrema* sp., while *Kurtzmaniella* sp. and *Wickerhamiella* sp. were found as single isolates. According to the findings, *Begonia obliqua*, which has six yeast species, *Rondeletia leucophylla*, which has five, and *Alcea rosea*, which has four, are the flowers that best depict the diversity of yeast (Fig. 1). Numerous yeast species linked to various flowers are revealed by the study, underscoring the ecological uniqueness or adaptability of distinct yeasts to specific floral niches. Interestingly, *Aureobasidium melanogenum* and *Metschnikowia koreensis* was isolated from several floral sources, indicating a wider host range, but other species, such as *C. tropicalis, Lachancea thermotolerans*, and *S. polymorphus* seemed to be exclusive to particular flowers. This dataset encourages more research on the ecological interactions between yeast and flowers and highlights the significance of floral biodiversity in determining yeast spread.

The presence of species such as *Starmerella floricola* and *Metschnikowia koreensis* is particularly noteworthy, as these are well-documented as common inhabitants of flowers globally (Hong et al., 2001; Lachance et al., 2001). The results indicate that certain floral species, namely *Begonia obliqua, Rondeletia leucophylla*, and *Alcea rosea*, host a greater diversity of yeast species compared to others. This differential yeast richness across floral sources may be attributed to a number of factors, including the chemical composition of the nectar, the physical structure of the flower, and the specific pollinator community associated with each plant (Jacquemyn et al., 2021).

The presence of genera like *Aureobasidium, Papiliotrema, Kurtzmaniella*, and *Wickerhamiella* further enriches the understanding of floral yeast communities in tropical regions (Kanpiengjai et al., 2023; Klaps et al., 2020; Lachance et al., 2001). These findings underscore the importance of geographical location and specific plant-microbe interactions in determining the composition of floral yeast communities. The study’s results lay a solid foundation for further research into the functional roles of these yeasts in the floral ecosystem.

### Selection of high carotenoid production potential in red yeast

In an initial screening, 36 yeast strains isolated from flowers which produced pigmented colonies cultured on YM agar were selected. These strains were further cultivated in 50 mL of YPD broth for 3 days. The results showed a wide range of growth, with optical density at 600 nm (OD_600_) varying from 1.18 to 8.71, and carotenoid production ranging from 12.10 to 70 mg/L (Supplementary Fig. S2). Based on these preliminary findings, five strains with the highest carotenoid production potential (43.55–70.50 mg/L of carotenoid content) were selected for further investigation: *Rhodotorula taiwanesis* SWU-FCT10, *Rhodotorula paludigena* SWU-FR02, *Aureobasidium melanogenu* SWU-FBN09, *Rhodotorula paludigena* SWU-FKT03, and *Aureobasidium melanogenum* SWU-FDS01.

**Figure 2:**
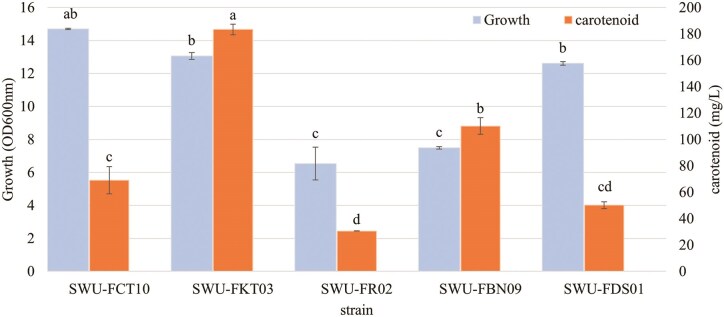
The growth and carotenoid concentration of the selected 5 yeast strains cultivated in 100 mL of YPD medium for 5 days. Growth (OD600 nm) is indicated on the left Y-axis and carotenoid concentration (mg/L) on the right Y-axis. Data are presented as mean ± SD (*n* = 3). Different lowercase letters above the bars indicate statistically significant differences between strains (p<0.05) according to Tukey’s post-hoc test. Statistical comparisons were performed separately for growth and carotenoid concentration.

These five yeast strains were cultured in a larger volume of 100 mL YPD broth for 5 days. As shown in Fig. 2, the results highlight a clear difference in both growth and carotenoid production among the strains. *R. paludigena* SWU-FKT03 shows the highest level of carotenoid production (183.3 ± 4.00 mg/L), followed by *A. melanogenu* SWU-FBN09 (110.5 ± 6.35 mg/L). Notably, while SWU-FKT03 has robust growth (OD_600_ = 13.06 ± 0.21), its growth level is statistically similar to that of *R. taiwanesis* SWU-FCT10 (OD_600_ = 14.70 ± 0.04) and *A. melanogenum* SWU-FDS01 (OD_600_ = 12.61 ± 0.11), which produce significantly less carotenoids. Conversely, *R. paludigena* SWU-FR02 demonstrates the lowest levels of both growth and carotenoid production (30.6 mg/L ± 0.05).

Interestingly, these results demonstrate that there is not always a direct correlation between cell growth and carotenoid production. For example, strain SWU-FCT10 exhibited the highest growth but produced low amounts of carotenoids (69.10 mg/L). In contrast, strains SWU-FKT03 and SWU-FBN09, with moderate growth, produced very high levels of carotenoids. This finding suggests that the factors influencing the production of secondary metabolites like carotenoids are strain-specific, rather than being solely dependent on the accumulated biomass.

The systematic screening of 36 isolates successfully identified *R. paludigena* SWU-FKT03 as an elite producer, yielding 183.3 mg/L of carotenoids in the initial secondary screening. The qualitative analysis by Thin Layer Chromatography revealed that the carotenoid extract from the selected yeast strain exhibited a retention factor (*Rf*) of 0.94. This value was identical to that of the authentic β-carotene standard run under the same conditions, indicating that the major carotenoid produced by this strain is β-carotene. This discovery is significant, as the *Rhodotorula* genus is widely recognized as a potent source of commercially valuable carotenoids, including β-carotene and torulene (Frengova & Beshkova, 2009; Wang et al., 2019). The potential application of *R. paludigena* strains has already been highlighted in aquaculture as a pigment enhancer in fish diets (Rekha et al., 2024). Therefore, based on its superior and efficient carotenoid synthesis, *R. paludigena* SWU-FKT03 was selected as a strong candidate for further process optimization.

### Optimization of high carotenoid production potential in red yeast

To identify the optimal nutritional conditions for carotenoid synthesis by *R. paludigena* SWU FKT03, a two-step optimization of the cultivation medium was performed. The effects of different carbon and nitrogen sources on cell growth (OD600nm) and volumetric carotenoid concentration (mg/L) were systematically investigated over a 120-h period. Then the determination of the most suitable concentration of carbon and nitrogen sources were performed.

#### Effect of carbon and nitrogen source selection

**Figure 3:**
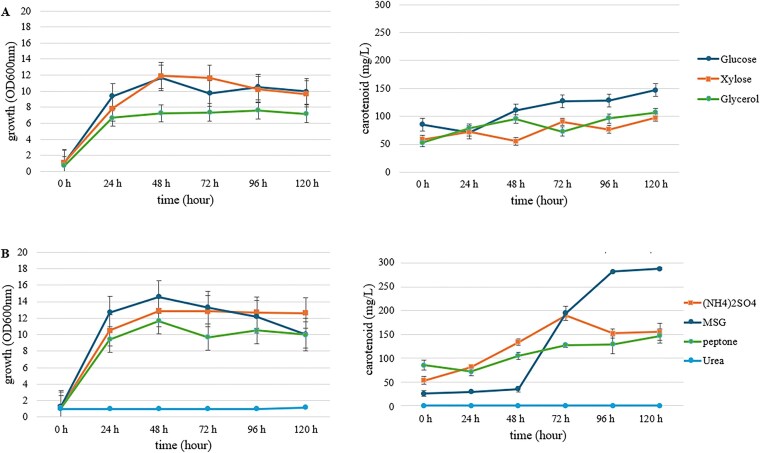
Growth and carotenoid concentration of *R. paludigena* SWU-FKT03 cultured in media with different carbon and nitrogen sources. (A) Effect of carbon sources (20 g/L) on growth and production. (B) Effect of nitrogen sources (adjusted nitrogen concentration equivalent to 20 g/L peptone) on growth and production. Data are presented as mean ± SD (*n* = 3).

The initial optimization step involved evaluating three carbon sources (glucose, xylose, glycerol) and four nitrogen sources ((NH_4_)₂SO_4_, MSG, peptone, urea), added to a basal medium containing 10 g/L yeast extract, for their effect on cell growth and carotenoid synthesis (Fig. 3).

The type of carbon source significantly influenced both the growth and carotenoid production profiles of SWU-FKT03 (Fig. 3A). In terms of cell density, xylose promoted the highest biomass, achieving a peak OD600 of approximately 11.92 ± 0.07 at 48 h. Glucose also supported robust growth, reaching a maximum OD600 of 11.92 ± 0.48 at 48 h. Glycerol was the least effective of the three, resulting in a lower peak biomass (OD600 = 7.58 ± 0.98 at 96 h).

Conversely, when assessing carotenoid production, glucose was identified as the superior carbon source. The carotenoid concentration in the glucose medium increased steadily throughout the fermentation, reaching the highest concentration of 147.03 ± 14.87 mg/L at 120 h. While xylose and glycerol supported moderate pigment synthesis, they yielded significantly lower final concentrations of 97.47 ± 4.96 mg/L and 106.38 ± 1.53 mg/L, respectively. Notably, the optimal carbon source for biomass accumulation (xylose) was not the optimal source for carotenoid production, highlighting a potential decoupling of primary growth and secondary metabolite synthesis.The selection of a nitrogen source had an even more pronounced effect on carotenoidogenesis (Fig. 3B). Among the four sources tested, monosodium glutamate (MSG), dramatically enhanced carotenoid production. After 48 h, the carotenoid concentration increased sharply, achieving a remarkable peak of 288.27 ± 0.04 mg/L at 96 h. MSG and ammonium sulfate ((NH_4_)₂SO_4_), supported good biomass-with MSG yielding the highest cell density overall (OD600 = 14.89 ± 0.20 at 48 h). Urea was found to be an unsuitable nitrogen source for this strain, as it supported neither significant growth nor carotenoid production.

In summary, these findings demonstrate that glucose and MSG are the optimal carbon and nitrogen sources, respectively, for maximizing carotenoid production by *R. paludigena* SWU-FKT03. The results further reinforce that conditions favoring maximal biomass are not necessarily congruent with those required for maximal secondary metabolite production, a critical consideration for subsequent process optimization.

#### Effect of glucose and MSG concentration

**Figure 4:**
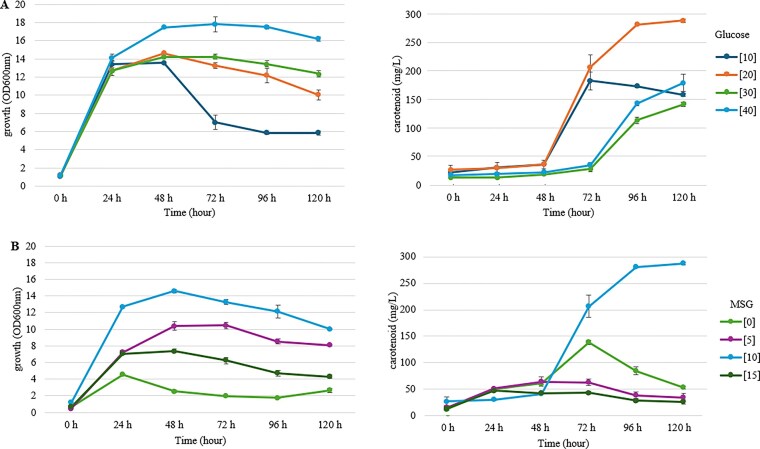
Growth and carotenoid concentration of *R. paludigena* SWU-FKT03 under varying nutrient concentrations. (A) Effect of different glucose concentrations (10–40 g/L) in a medium containing a fixed concentration of MSG (10 g/L) and yeast extract (10 g/L). (B) Effect of different MSG concentrations (0–15 g/L) in a medium containing a fixed concentration of glucose (20 g/L) and yeast extract (10 g/L). Data are presented as mean ± SD (*n* = 3).

Subsequently, the effects of nutrient concentration on the fermentation profile were investigated (Fig. 4). The optimal concentration of glucose was determined, alongside an investigation into the concentration-dependent effects of the potent organic nitrogen source, MSG.

As shown in Fig. 4A, cell growth was directly proportional to the initial glucose concentration, with 40 g/L yielding the highest biomass (OD_600_₀₀ =17.80 ± 0.22) in 72 h. However, carotenoid production did not follow the same trend. An initial glucose concentration of 20 g/L was found to be optimal, resulting in the highest carotenoid concentration of 288.27 ± 2.86 mg/L at 120 h. Notably, increasing the glucose concentration to 40 g/L led to a significant reduction in carotenoid concentration (178.93 ± 14.67 mg/L), despite supporting higher cell density, suggesting a potential inhibitory or catabolite repression effect at elevated substrate levels.

The investigation of the optimum concentration of MSG revealed very specific results (Fig. 4B). A concentration of 10 g/L of MSG was overwhelmingly superior for carotenoid production. Both lower (5 g/L) and higher (15 g/L) concentrations of MSG were strongly detrimental to pigment synthesis, resulting in produces below 150 mg/L, even though 10 g/L and 15 g/L supported robust biomass. This indicates that the nitrogen concentration for maximal carotenoidogenesis exists within a narrow optimal range. However, It is worth noting that the medium was not buffered. Consequently, the metabolism of high concentrations of MSG (15 g/L) could lead to significant pH fluctuations during fermentation, which may have contributed to the reduced carotenoid synthesis observed compared to the 10 g/L condition.

Collectively, this nutritional optimization successfully enhanced carotenoid production significantly, identifying the optimal conditions within the tested parameters. Through systematic screening of carbon and nitrogen sources was followed by concentration optimization, a medium containing 20 g/L of glucose and 10 g/L of MSG was identified as optimal, representing a higher carotenoid concentration of 288.27 mg/L from 183.3 mg/L in nonoptimum conditions.

A key finding of this study is the decoupling of biomass accumulation from carotenoid production. This is a well-documented phenomenon in secondary metabolism, where pigment synthesis is often triggered under specific nutrient-limited or stress conditions, rather than during maximal growth (Aizpuru & González-Sánchez, 2024). Nutritional optimization of *Rhodotorula paludigena* SWU-FKT03 identified an optimal medium of 20 g/L glucose and 10 g/L MSG, corresponding to a C/N ratio of approximately 14. This specific balance is crucial for triggering a metabolic shift from biomass accumulation to pigment synthesis, a process that was otherwise inhibited by higher glucose levels due to carbon catabolite repression (Braunwald et al., 2013; Mata-Gómez et al., 2014; Sereti et al., 2024a). The observed decrease in carotenoid production at higher glucose concentrations (40 g/L), despite increased biomass, strongly suggests the occurrence of carbon catabolite repression, a regulatory mechanism that prioritizes growth over secondary metabolite synthesis (Gancedo, 1998; Ruiz-Villafán et al., 2022). Ultimately, these findings underscore that maximizing carotenoid production is not merely about providing excess nutrients, but about achieving a precise metabolic balance that redirects carbon flux from proliferation to pigment synthesis.

### Fermentation performance and economic potential

Under optimized shake-flask conditions, *R. paludigena* SWU-FKT03 showed outstanding carotenoid biosynthesis (Table 1), reaching a final titter of 288.27 mg/L, a specific content of 119.06 mg/g DCW, a volumetric productivity of 2.40 mg/L/h, and a substrate yield of 14.41 mg/g glucose. Compared with a YPD medium, optimization with glucose and MSG resulted in nearly a three-fold increase in carotenoid content per biomass. Interestingly, biomass accumulation decreased, suggesting that the MSG source redirected metabolic flux from growth toward secondary metabolite synthesis, consistent with reports that nitrogen supplementation can modulate the mevalonate pathway and enhance precursor availability for carotenoid biosynthesis (Naz et al., 2020; Sereti et al., 2024a).

**Table 1:** Comparison of fermentation performance and carotenoid production of *R. paludigena* SWU-FKT03 with other red yeast strains.

Species	Carbon sources	Carbon utilization (g/L)	Cultivation time (h)	Biomass (g/L)	Carotenoid production (mg/L)	Carotenoid content (mg/g DCW)	Carotenoid yield (mg/g carbon)	Carotenoid productivity (mg/L/h)	References
*R. paludigena* SWU-FKT03									
- Before optimized	Glucose (YPD medium)	20	120	4.02	183.30	45.6	9.17	1.53	This study
- Optimized condition	Glucose + MSG	20	120	2.42	288.27	119.1	14.41	2.40	This study
*R. glutinis*	molasses	20	150	4.4	125.00	28.4	6.25	0.83	(Aksu & Eren, 2007)
*R. gracilis* ATCC 10 788	Glycerol	30	96	21.8	6.24	0.3	0.21	0.07	(Kot et al., 2020)
*R. mucilaginosa* M22	Glycerol	20	120	23.9	5.45	0.2	0.27	0.05	(Allahkarami et al., 2021)
*R. mucilaginosa* RGM42	Glucose	20	72	9.5	5.70	0.6	0.29	0.08	(Fonseca et al., 2025)
*Rh. kratochvilovae* Y-42	hydrolyzed cheese whey	50	63	14.6	36.35	2.8	0.72	0.58	(Sereti et al., 2024b)
*Rh. toruloides* C23	Glucose	120	144	26.9	21.42	0.8	0.18	0.21	(Xue et al., 2023)

The performance of SWU-FKT03 markedly surpasses previously reported red yeasts in terms of final titer, specific content, and productivity. Regarding volumetric production, *R. glutinis* TISTR 5159 produced 135.25 mg/L from crude glycerol (Saenge et al., 2011) and *R. glutinis* produced 125.00 mg/L from molasses while *R. paludigenum* 2663 yielded only 2.21 mg/L from on sucrose (Sereti et al., 2024a).

In terms of cellular accumulation, the specific carotenoid content of SWU-FKT03 was exceptionally high at 119.06 mg/g DCW. This value exceeds the 15.39 mg/g DCW reported for *R. paludigena* CM33 (Kaewda et al., 2025) and far greater than the 0.2–28.4 mg/g observed from other studies (Table 1). Likewise, the productivity of 2.40 mg/L/h outperformed most references, including *R. glutinis* (0.83 mg/L/h; Aksu & Eren, 2007), *Rh. kratochvilovae* Y-42 (0.58 mg/L/h; Sereti et al., 2024b), and *R. mucilaginosa* strains that rarely exceeded 0.05–0.08 mg/L/h (Allahkarami et al., 2021; Fonseca et al., 2025). Collectively, these findings establish SWU-FKT03 as a superior carotenoid-producing yeast.

Economic evaluation further demonstrated the industrial potential of this strain (Table 2). Replacing peptone with MSG reduced raw material costs by more than 80% while simultaneously increasing yield by 57%. Given that peptone is a major cost driver in microbial fermentation, its substitution with a low-cost nitrogen source significantly improves process feasibility. This dual advantage—enhanced biosynthetic efficiency and reduced media cost—positions *R. paludigena* SWU-FKT03 as an excellent candidate for scale-up.

**Table 2:** Summary of the raw material cost calculation for the carotenoid production from YPD and Modified YPD by *R. paludigena* SWU-FKT03 (plant capacity: 10 kt carotenoid/y).

Media cultivation	Raw material	Amount (g/L)	Amount (t/y)	Unit cost^a^ (USD/t)	Total cost (M-USD/y)	%
YPD	Yeast extract	10	546	5 750	3.14	19
183.3 g/L Carotenoid production	Peptone	20	1 091	12 000	13.09	77
	Glucose	20	1 091	615	0.67	4
				Total	16.90	100
Modified YPD	Yeast extract	10	347	5 750	1.99	69
288.27 g/L Carotenoid production	MSG	10	347	1 350	0.47	16
	Glucose	20	694	615	0.43	15
				Total	2.89	100

a The unit cost is based on the midpoint of the estimated price range from the US market assessment for Q1–Q2 2025.

Together, these results identify SWU-FKT03 as a robust, nonengineered yeast strain with strong potential for sustainable, large-scale carotenoid production. Future studies should investigate the use of agro-industrial residues such as soybean meal or corn steep liquor to further reduce media costs and evaluate omics-based insights into MSG-mediated regulation of carotenoid metabolism.

## Conclusion

This study successfully demonstrated the rich and largely untapped biodiversity of yeasts associated with Thai flowers, leading to the discovery of a potent new carotenoid producer. Through a systematic screening of 36 pigmented isolates, *Rhodotorula paludigena* SWU-FKT03 was identified as a superior strain. A subsequent two-stage nutritional optimization significantly enhanced its productive capabilities, with a medium containing 20 g/L of glucose and 10 g/L of monosodium glutamate boosting the final carotenoid titer by 1.5-fold to a remarkable 288.27 mg/L. The exceptional performance of this nonengineered strain was further confirmed by its high specific carotenoid content (119.1 mg/g DCW) and volumetric productivity (2.40 mg/L/h), metrics that are highly competitive with and often exceed those of previously reported wild-type strains. These findings not only contribute a novel, high-performance biocatalyst to the field of biotechnology but also validate bioprospecting from unique floral ecosystems as a potential strategy for discovering industrially valuable microorganisms. The robust performance of *R. paludigena* SWU-FKT03 establishes it as a promising candidate for the sustainable, large-scale production of natural carotenoids.

## Supplementary Material

kuag003_Supplemental_File

## Data Availability

The data underlying this article will be shared on reasonable request to the corresponding author.
