# Supplementary material for: Exploration of yeast biodiversity from Thai flowers and optimization of carotenoid production by a promising isolate
Source: J Ind Microbiol Biotechnol. 2026 Jan 8;53:kuag003. doi: 10.1093/jimb/kuag003 (PMC12883991; doi:10.1093/jimb/kuag003)
Supplement: kuag003_Supplemental_File [file kuag003_supplemental_file.pdf]

Supplementary Figure

Exploration of Yeast Biodiversity from Thai Flowers and Optimization of Carotenoid Production by a Promising Isolate

Pirapan Polburee[1,*], Thippawan Kodpan[1], Krittawan Tondee[1], Nontakorn Wimoolchat[1]

[1]Department of Microbiology, Faculty of Science, Srinakharinwirot University, 114 Sukhumvit 23, Bangkok 10110, Thailand

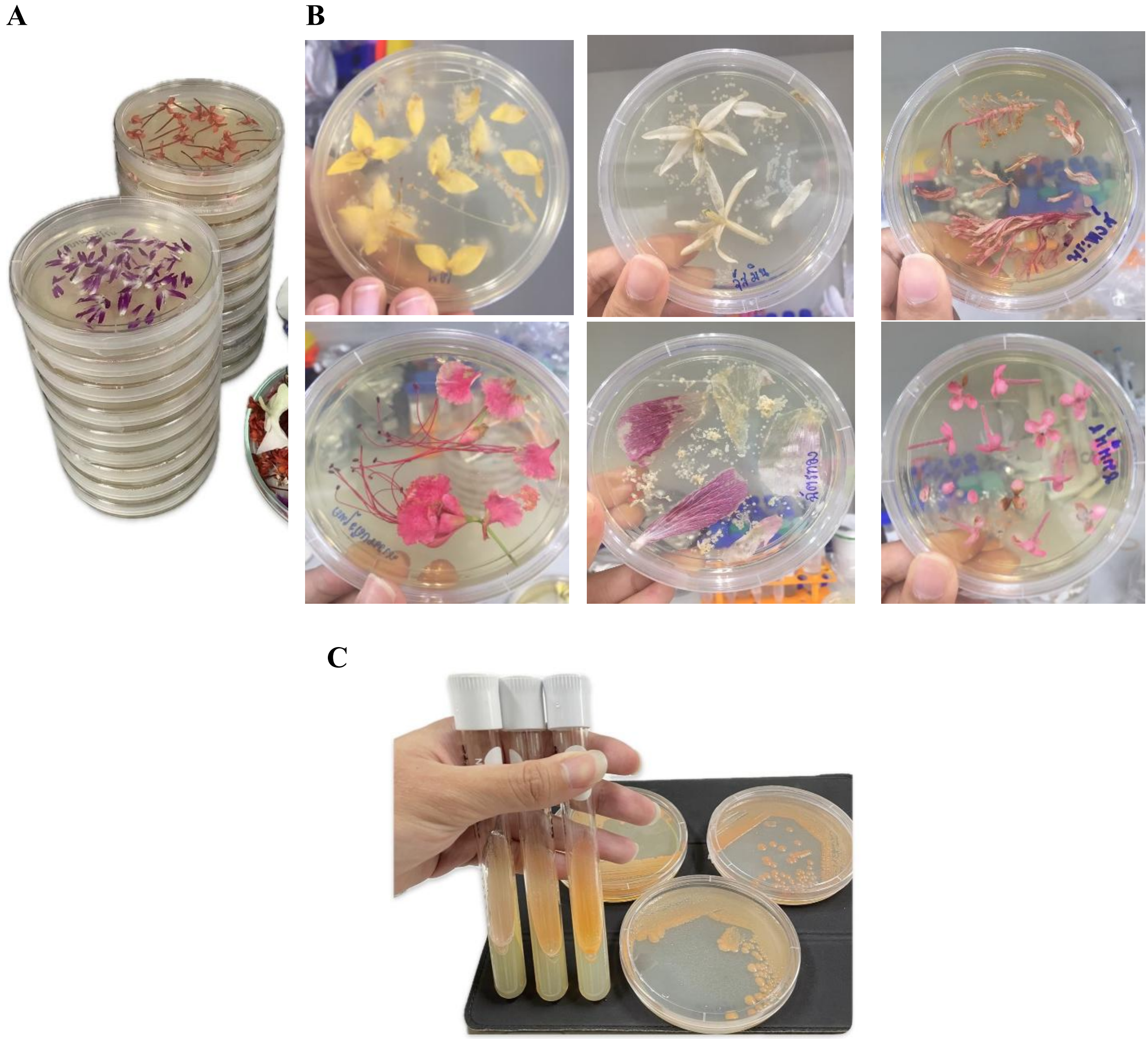

Supplementary Figure S1. Procedure for yeast isolation from flowers.
(A) Flower samples were placed directly onto YM medium (direct plating method).
(B) Appearance of yeast colonies growing from various flower samples after incubation.
(C) Pure cultures of selected red yeast strains maintained on slants and agar plates.

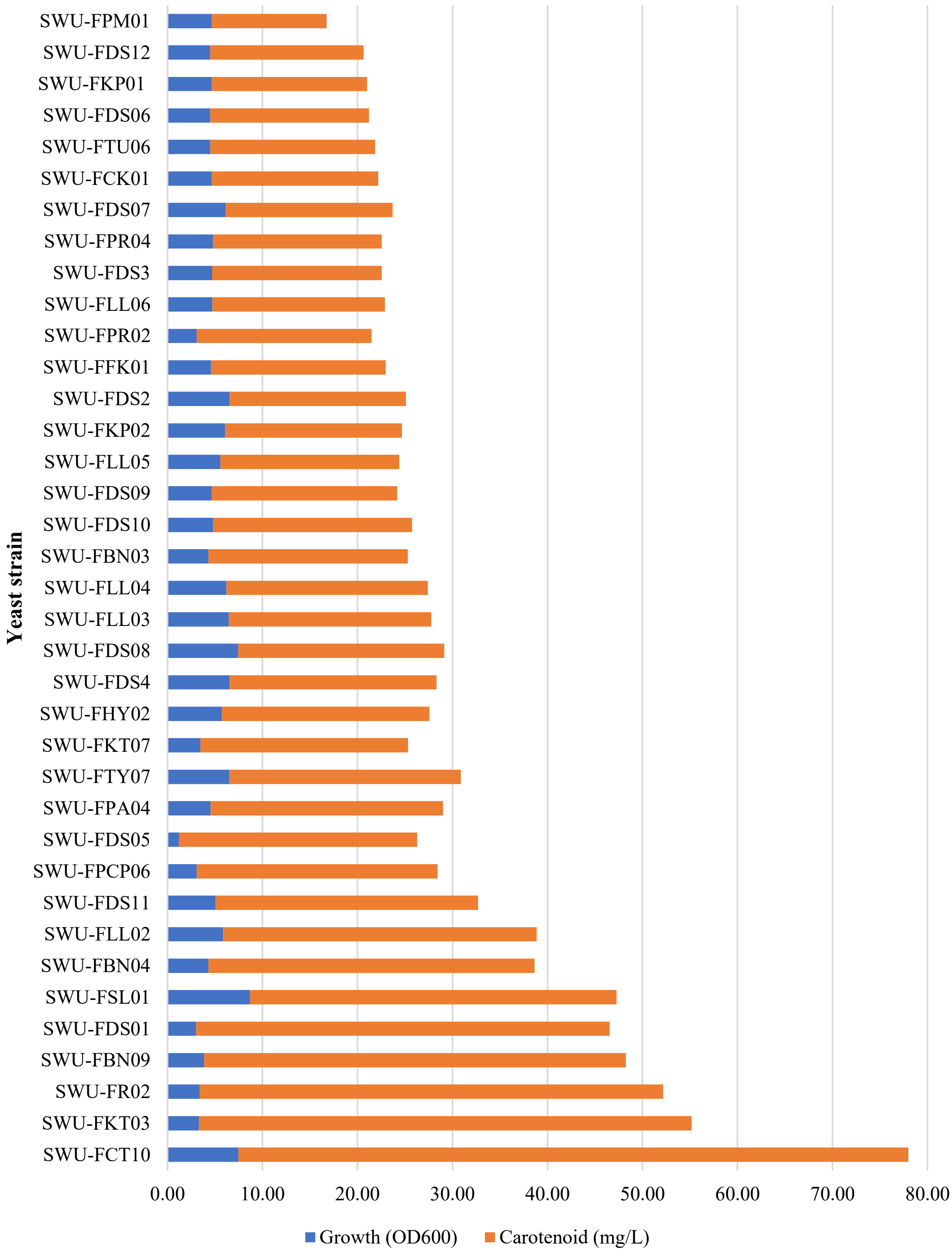

Supplementary Figure S2. Screening of 36 yeast strains isolated from flowers for growth and carotenoid production. Strains were cultivated in YPD broth for 3 days. The blue bars represent cell growth measured by optical density at 600 nm (OD600) and the orange bars represent carotenoid concentration (mg/L).
